# Rule Extraction From Binary Neural Networks With Convolutional Rules for Model Validation

**DOI:** 10.3389/frai.2021.642263

**Published:** 2021-07-21

**Authors:** Sophie Burkhardt, Jannis Brugger, Nicolas Wagner, Zahra Ahmadi, Kristian Kersting, Stefan Kramer

**Affiliations:** ^1^ Institute of Computer Science, Johannes Gutenberg University of Mainz, Mainz, Germany; ^2^ Centre for Cognitive Science, and Hessian Center for Artificial Intelligence, Department of Computer Science, TU Darmstadt, Darmstadt, Germany

**Keywords:** k-term DNF, stochastic local search, convolutional neural networks, logical rules, rule extraction, interpretability

## Abstract

Classification approaches that allow to extract logical rules such as decision trees are often considered to be more interpretable than neural networks. Also, logical rules are comparatively easy to verify with any possible input. This is an important part in systems that aim to ensure correct operation of a given model. However, for high-dimensional input data such as images, the individual symbols, i.e. pixels, are not easily interpretable. Therefore, rule-based approaches are not typically used for this kind of high-dimensional data. We introduce the concept of first-order convolutional rules, which are logical rules that can be extracted using a convolutional neural network (CNN), and whose complexity depends on the size of the convolutional filter and not on the dimensionality of the input. Our approach is based on rule extraction from binary neural networks with stochastic local search. We show how to extract rules that are not necessarily short, but characteristic of the input, and easy to visualize. Our experiments show that the proposed approach is able to model the functionality of the neural network while at the same time producing interpretable logical rules. Thus, we demonstrate the potential of rule-based approaches for images which allows to combine advantages of neural networks and rule learning.

## 1 Introduction

^1^Neural Networks (NNs) are commonly seen as black boxes, which makes their application in some areas still problematic (e.g., in safety-relevant applications or applications in which DL is only intended to support a human user). Logical statements are however easier to process by humans than the main building blocks of NNs (e.g., nonlinearities, matrix multiplications, or convolutions). In general, learning of logical rules cannot be done using gradient-based algorithms as they are not differentiable. Even if we find rules that exactly describe a neural network, they might still be too complex to be understandable. In this paper we propose convolutional rules for which the complexity is not related to the dimensionality of the input but only to the dimensionality of the convolutional filters. Thereby we aim to combine advantages from two fields: We make use of the NN’s ability to handle high-dimensional data and we allow for model validation, not just through visualization and subjective assessment, but through rigorous logical rules.

It is a wide-spread belief that shorter rules are usually better than longer rules, a principle known as Occam’s razor. This common assumption was recently challenged again by Stecher et al. (2016), who revive the notion of so-called *characteristic rules* instead. As they show, shorter rules are often discriminative rules that help to differentiate different output classes, but are not necessarily descriptive of the data. In this work, we can confirm this observation and show how characteristic rules are produced for high-dimensional input data such as images.

Specifically, based on recent developments in deep learning with binary neural networks (BNNs) (Hubara et al., 2016), we propose an algorithm for *decompositional rule extraction* (Andrews et al., 1995), called *Deep Convolutional DNF Learner (DCDL)*. A BNN takes binary input and only produces binary outputs in all hidden layers as well as the output layer. Some BNNs also restrict weights to be binary, however, this is not necessary for our approach. We then approximate each layer using rules and combine these rules into one rule to approximate the whole network. As our empirical results show, this allows for better approximation than with an approach that considers the neural network to be a black box—a so-called *pedagogical rule extraction* approach.

Moreover, we show how the convolutional rules are used to visualize what the network has learned, a feature not available for other types of logical rules that would not lead to any meaningful visualization.

As parallel developments Narodytska et al. (2020); Jia and Rinard (2020) show, the use of binary neural networks for model validation is a promising research direction, because SAT-solvers can be employed more efficiently in these networks than Satisfiability Modulo Theory (SMT) solvers that are usually applied on non-binary networks. Thus, our work can be seen as complementary to theirs, and we focus more on the visualization of the rules and the approximation of the convolutional filters here although the final goal of eventually applying SAT-solvers or related techniques in order to verify model functionality is the same.

To sum up, our contributions are as follows:

• We formally define first-order convolutional rules (Section 3.1) to describe a neural network using rules that are less complex than the original input.
• We show that the decompositional rule extraction approach performs better than the approach that considers the network as a black box in terms of approximating the functionality of the neural network.
• We show how the convolutional rules produce characteristic visualizations of what the neural network has learned (although we want to emphasize that our goal is not to explain individual classifications).
• To our knowledge, for the first time we show how logical rules can be used on high-dimensional data such as images and still yield interpretable results.

We proceed as follows. We start off by touching upon related work on binary neural networks, rule extraction, and interpretability in convolutional networks in Section 2. Deep Convolutional DNF Learner (DCDL), consisting of the specification of first-order convolutional rules and the SLS algorithm, is then introduced in Section 3. Our experimental results on similarity, accuracy, and the visualization are presented and discussed in Section 4.

## 2 Related Work

Our work builds upon binary neural networks, rule extraction, and visualization of convolutional neural networks.

### 2.1 Binary Neural Networks

Binary Neural Networks (BNNs) are neural networks that restrict the target of activation functions and weights to binary values {−1,1}. The original motivation for BNNs is to reduce the memory footprint of NNs and accelerate inference (Hubara et al., 2016). BNNs can be stored more efficiently because binary values can be stored in 1-bit instead of 32 bits or 64 bits. Also, binary representations can avoid computationally expensive floating-point operations by using less expensive, bitwise operations, leading to a speed-up at inference time (Rastegari et al., 2016).

However, by construction, the activation functions of BNNs lack differentiability and have less representational power due to their limitation to binary output values. Research on BNNs focuses on alleviating these two limitations. A breakthrough for BNNs was the straight-through estimator (STE) introduced in Hinton’s lectures (Hinton, 2012). The STE calculates the gradient of the Heaviside step function *H* as if it was the identity function. By using the STE in combination with the sign function B(x)=2⋅H(x)−1 instead of *H*, Hubara et al. (2016) demonstrate the general capabilities of BNNs. They maintain real-valued weights while using binarized weights only for inference and calculation of gradients. Training updates are applied to the real-valued weights. They adapt the STE to better fit *B* by clipping the identity function at −1 and 1 (Clipped STE). Nevertheless, the sole usage of the (Clipped) STE does not compensate for the lack of representational power of BNNs. Therefore, further improvements were proposed (Rastegari et al., 2016; Lin et al., 2017).

### 2.2 Rule Extraction

Rule extraction algorithms are commonly divided into decompositional and pedagogical approaches (Andrews et al., 1995). Pedagogical (or model-agnostic) approaches view the neural network as a black box and approximate its global function using rules, whereas decompositional methods make use of the individual components of the network in order to construct the set of rules. Our work follows a decompositional approach, allowing a better approximation as compared to the pedagogical approach that we compare to in our experiments.

State-of-the-art pedagogical approaches include validity interval analysis (VIA), sampling, and reverse engineering. VIA (Thrun, 1993, Thrun, 1995) searches for intervals in the input data within which the NN produces the same output. The found intervals can be transformed into rules. Approaches using sampling (Craven and Shavlik, 1995; Schmitz et al., 1999; Taha and Ghosh, 1999; Sethi et al., 2012) try to let the NN label especially important instances in order to learn better rules. For instance, sampling can be beneficial to learn rules on parts of the unknown label function which are not covered well by the training instances. The reverse engineering approach by Augasta and Kathirvalavakumar (2011) prunes the NN before the rules are extracted. As a result, the extracted rules are more comprehensible. Setiono and Leow (2000) use a similar technique to identify the relevant perceptrons of a NN.

Among others, decompositional algorithms use search techniques to find input combinations that activate a perceptron (Fu, 1994; Tsukimoto, 2000). Some search techniques provably run in polynomial time (Tsukimoto, 2000). More recently, Zilke et al. (2016) proposed an algorithm that extracts decision trees per layer which can be merged into one rule set for the complete NN. González et al. (2017) improve on this algorithm by polarizing real-valued activations and pruning weights through retraining. Both rely on the C4.5 (Quinlan, 2014) decision tree algorithm for rule extraction. Kane and Milgram (1993) use a similar idea but cannot retrain an arbitrary already existing NN. Right from the beginning, they train NNs having (almost) binary activations or perceptrons, which are only capable of representing logical AND, OR, or NOT operations. Rules are extracted by constructing truth tables per perceptron.

Unfortunately, the existing decompositional rule extraction algorithms have no principled theoretical foundations in computational complexity and computational learning theory. The runtime of the search algorithm developed by Tsukimoto (Tsukimoto, 2000) is a polynomial of the number of input variables. However, this holds only if the number of literals that constitute a term of an extracted logical rule is fixed.

The other presented search algorithms exhibit an exponential runtime. Additionally, all mentioned search algorithms lack the possibility to fix the number of terms per extracted logical rule. Although the more recent decision tree-based approaches (Zilke et al., 2016) apply techniques to reduce the complexity of the extracted rules, they cannot predetermine the maximum model complexity. The only strict limitation is given by the maximum tree depth, which corresponds to the maximum number of literals per term of a logical rule. In general, the C4.5 algorithm does not take complexity restrictions into account while training. Directly being able to limit complexity, in particular the number of terms of a disjunctive normal form (DNF) in rule extraction, is desirable to fine-tune the level of granularity of a requested approximation.

In addition to pedagogical and decompositional approaches, there are approaches for local explanations, which explain a particular output, and visualization Ribeiro et al. (2016). Also, there are approaches that create new models that are assumed to be more interpretable than the neural network (Odense and Garcez, 2020). As this is not our goal, we focus on the decompositional approach in our work.

### 2.3 Convolutional Networks and Interpretability

Concerning the interpretability of convolutional neural networks, existing work can be divided into methods that merely visualize or analyze the trained convolutional filters (Simonyan et al., 2013; Zeiler and Fergus, 2014; Mahendran and Vedaldi, 2015; Zhou et al., 2016) and methods that influence the filters during training in order to force the CNN to learn more interpretable representations (Hu et al., 2016; Ross et al., 2017; Stone et al., 2017). Our work can be situated in between those two approaches. While we do change the training procedure by forcing the CNN to use binary inputs and generating binary outputs, we also visualize and analyze the filters after training by approximating the network with logical rules.

In order to make convolutional neural networks more interpretable, Zhang et al. (2018) propose a method to learn more semantically meaningful filters. This method prevents filters from matching several different object parts (such as the head and the leg of a cat) and instead leads to each filter only detecting one specific object part (e.g., only the head), thus making the filters more interpretable. In contrast, our approach allows for different object parts being represented in one filter, but then uses the approximation with rules to differentiate between different object parts. One term in a k-term DNF (see Section 3.1 for the definition of k-term DNFs) might correspond to one specific object part.

While these approaches are looking at the interpretability of CNNs, in essence they all resort to local explanations and visualizations of network outputs. Rule learning provides a global approach that is much more precise and exactly explains how a certain output was generated. Apart from one recent attempt for propositional rules (Bologna, 2019) we are not aware of any other work that learns logical rules from CNNs. In this work, we argue that predicate logic is more suitable for logical rule extraction from CNNs.

## 3 Deep Convolutional DNF Learner

Our approach draws inspiration from recent work on binary neural networks, which are able to perform almost on par with non-binary networks in many cases (Lin et al., 2017; Liu et al., 2018). These networks are built of components that can provably be transformed into logical rules. However, for this work we only used the Clipped STE as it appeared sufficient for smaller datasets and networks that were used in our experiments. For the CIFAR dataset there could be an advantage to adding scaling. Further improvements of the binary network architecture are therefore possible, also batch normalization might improve performance in some cases, but this was not our main focus.

The basic building blocks of any NN are variants of perceptrons. To ensure that a perceptron can be represented by a logical expression, we need to restrict the input as well as the output to binary values. This allows to transform perceptrons into truth tables. For hidden layers, we have to ensure that the output is binary leading to binary input for subsequent layers. For the input layer, we need to establish a binarization mechanism for categorical and a discretization mechanism for continuous features. The binarization of a categorical feature with *n* possible values is done in a canonical way by expanding it into *n* binary features. For the discretization of continuous features we use dithering. In particular, we use the Floyd-Steinberg algorithm^2^ to dither the gray scale images to black and white images and dither the individual channels of RGB images. We tested Floyd-Steinberg, Atkinson, Jarvis-Judice-Ninke, Stucki, Burkes, Sierra-2-4a, and Stevenson-Arce dithering,^3^ which are based on error diffusion, and found no statistically significant differences in the performance of the neural network using a corrected resampled *t*-test (Nadeau and Bengio, 2003).

A standard perceptron using the heaviside-function satisfies the requirement of binary outputs, but is not differentiable (unless using the delta-distribution). However, the straight-through estimator (Bengio et al., 2013) calculates gradients by replacing the heaviside-function with the identity function and thus allows to backpropagate the gradients.

To be able to regularize complexity, we employ an adaptation of the stochastic local search (SLS) algorithm (Rückert and Kramer, 2003) to extract logical expressions with *k* terms in disjunctive normal form (k-term DNF). SLS can be parameterized with the number of terms to learn and thereby limit the maximum complexity. As the SLS algorithm is run after an NN has been trained, we do not limit the complexity at training time. Hinton et al. (2015) have already shown that this can be advantageous.

Convolutional Neural Networks (CNNs) are important architectures for deep neural networks (Mnih et al., 2013; Gehring et al., 2017; Poplin et al., 2018). Although convolutional layers can be seen as perceptrons with shared weights, logical expressions representing such layers need to be invariant to translation, too. However, logical expressions are in general fixed to particular features. To overcome this issue, we introduce a new class of logical expressions, which we call *convolutional logical rules*. Those rules are described in relative positions and are not based on the absolute position of a feature. For inference, convolutional logical rules are moved through data in the same manner as convolutional filters. This ensures interpretability and lowers the dimensionality of extracted rules.

Pooling layers are often used in conjunction with convolutional layers, and max-pooling layers guarantee binary outputs given binary inputs. Fortunately, binary max-pooling can easily be represented by logical expressions in which all input features are connected by a logical OR. The algorithms for training and testing DCDL are summarized in Algorithms 1, 2.

Algorithm 1

1: **procedure** Train DCDL (number of layers *L*).
2:**** ϕ ← ∅
3: ** for** layer l=1,…,L **do**.
4: **  if** Convolutional layer **then**.
5:**** ψ←∅
6: **   for** Convolutional filter *f* **do**.
7:**** $\psi_{f}\leftarrow$rule learner on input and output of NN for this filter.
8:**** $\psi \;\leftarrow \;\psi \;\cup \;\psi_{f}$
9:**** ϕ ← ϕ ∪(l,ψ)
10: **  else if** Max pooling **then**.
11: **   **No training required.
12: **  else if** Dense **then**.
13:**** ψ←rule learner on input and output of NN.
14:**** ϕ ← ϕ ∪ (l,ψ)
** return** ϕ

Algorithm 2

1: **procedure** Test DCDL (input data, trained rule learner models ϕ).
2:**** **input** ←input data.
3: ** for** ψ∈ϕ **do**.
4: **  if** Convolutional layer **then**.
5:**** λ←∅
6: **   for** rule $\psi_{i}\in \psi$ **do**.
7:**** $\lambda_{i}\leftarrow$evaluate rule $\psi_{i}$ on input.
8:**** $\lambda \leftarrow \lambda \cup \lambda_{i}$
9: **  else if** Max pooling **then**.
10: **   for** Output neuron **do**.
11: **    **Combine values in each pool with or-operation.
12: **  else if** Dense **then**.
13:**** λ←evaluate ψ on input.
14: **  **input←λ
** return** prediction of DCDL λ

### 3.1 Introduction of First-Order Convolutional Rules

This section provides the formal underpinnings and the introduction of the convolutional rules. We start with propositional k-DNF formulas and then move on to use first-order logic to take advantage of variable assignments (variables representing relative pixel positions) as we shift the filter across the image.

A k-term DNF combines Boolean variables $\left\{x_{0},x_{1},\ldots ,x_{n-1}\right\}$ as *k* disjunctions of conjunctions

$$\overset{k-1}{\underset{i=0}{\vee }}\overset{m_{i}}{\underset{j=0}{\wedge }}x_{i,j},$$ (1)

where $x_{i,j}\in \left\{x_{0},\neg x_{0},x_{1},\neg x_{1},\ldots ,x_{n-1},\neg \neg x_{n-1}\right\}$ and $m_{i}\in \left\{0,1,\ldots ,n-1\right\}$. An example with k=3 and 3 input variables could look like (*x*_1_ ∧ *x*_2_) ∨ (*x*_3_) ∨ (¬*x*_1_ ∧ ¬*x*_2_).

In general, a rule is described in relation to a fixed set of input variables. Unfortunately, for image data this is not sufficient. The success of CNNs in image classification arguably stems from the translation invariance of filters. Thus, we propose that logical rules for image classification need to be invariant to translation as well. In the following we assume all pixels are binary.

Using propositional logic, the definition of convolutional logical rules is relatively complex. For a first-order convolutional logical rule definition the rule itself is straightforward and the complexity is shifted to the definition of the predicates and the environment with respect to which the predicates are evaluated. Due to the variability of the environment that is inherent in first-order logic, we can naturally account for the translation invariance of the rule. In other words, the rule stays the same, only the mapping of the variables to the concrete values in the Universe is changed as we move the rule over the image. Propositional logic on the other hand does not have variables and is thus not amenable to the translational invariance.

A k-term first-order convolutional logical rule (FCLR) is defined as follows: We define our model ℳ as the tuple (ℱ,P) consisting of a set of functions ℱ and a set of predicates P (Huth and Ryan, 2004). For a non-empty set *U*, the Universe of concrete values, each predicate P∈P is a subset $P^{ℳ}\subseteq U^{a}$ of tuples over *U*, where *a* is the number of arguments of predicate *P*. The Universe *U* of concrete values is defined as the set of concrete pixels in the image $p_{1},\ldots ,p_{n}\in \left\{0,1\right\}$.

A *first-order convolutional logical rule* with one term is now defined as

$$\phi_{conv}:=\exists x_{1},\ldots ,x_{a}:P_{conv}\left(x_{1},\ldots ,x_{a}\right),$$ (2)

where $P_{conv}\in P$ is the convolutional predicate and *a* is the size of the convolutional filter or the number of elements in the filter matrix. The convolutional predicate is defined as

$$P_{conv}:=\left\{\left(u_{1},\ldots ,u_{a}\right)|u_{i}\text{ are consecutive pixels in accordance}\text{with the convolutional filter}\right\}.$$ (3)

In order to evaluate our convolutional rule, we now need to specify the environment *l* (the look-up table) (Huth and Ryan, 2004) with respect to which our model satisfies (or not) the convolutional rule, i.e. $ℳ|{=}_{l}\phi$. The environment depends on the position at which we evaluate our convolutional rule. Evaluating the rule at position *t* means that the variables $x_{1},\ldots ,x_{a}$ are mapped to the corresponding pixels in the input image, $l_{t}\left[x_{1}\to p_{t}\right],l_{t}\left[x_{2}\to p_{t+1}\right],\ldots$, where $p_{1},\ldots ,p_{n}$ are the input image pixels (for 2D images, the indices have to be adjusted to account for the change to the next line, for simplicity, here the indices correspond only to 1D input). Thus, we are able to evaluate the convolutional rule in Eq. 2 at each position of the image.

Similar to the *k*-terms in DNF, we have *k* convolutional predicates, one for each of the *k* terms. Therefore, we can expand Eq. 2 to

$$\phi_{conv}:=\exists x_{1},\ldots ,x_{a}:P_{conv}^{\left(1\right)}\left(x_{1},\ldots ,x_{a}\right)\vee \ldots \vee P_{conv}^{\left(k\right)}\left(x_{1},\ldots ,x_{a}\right).$$ (4)

This concludes the definition of first-order convolutional rules.

#### 3.1.1 Example

**FIGURE 1:**
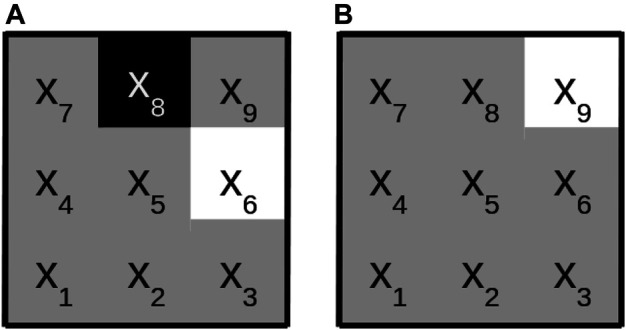
Graphical representation of the logical formula $P_{\text{conv}}^{\left(1\right)}\left(x\right)\vee P_{\text{conv}}^{\left(2\right)}\left(x\right)$, where $P_{\text{conv}}^{\left(1\right)}\left(x\right):=\left\{\left(x_{1},\ldots ,x_{m}\right)|x_{6}\wedge -x_{8}\text{ is true}\right\}$ and $P_{\text{conv}}^{\left(2\right)}:=\left\{\left(x_{1},\ldots ,x_{m}\right)|x_{9}\text{ is true}\right\}$. The first convolutional predicate is displayed on the **(A)**, the second one on the **(B)**. Variables which have to be true in order for the predicate to evaluate to true are marked white. Variables which have to be false in order for the predicate to evaluate to true are marked black. Variables that have no influence on the evaluation are marked gray.

The logical rules found by SLS may be displayed graphically, if the input for SLS is image data. For each image position *t* the variables of the convolutional predicates are mapped to the appropriate pixels using environment $l_{t}$. Thereby, each term of the k-term DNF can be visualized as an image. The whole convolutional rule can be output as a series of *k* gray scale images by displaying positive literals as white, negative literals as black and literals that do not influence the truth value as gray. Figure 1 shows an example of such a visualization with a rule that has two convolutional predicates and a filter size of 3×3.

### 3.2 Stochastic Local Search

We implemented the SLS rule learner^4^ and extended it for the purpose of pedagogical rule extraction (Algorithm 3). Since we apply SLS to predictive tasks, we adjust SLS to return the candidate that achieved the lowest score on the validation set (line 8). Scores used for the decision rule are still calculated on the training set. Calculation of scores is computationally expensive and SLS needs to evaluate the decision rule in every iteration. Therefore, we calculate scores batchwise. We introduce an adaptation that is theoretically motivated. One can always correct a term that falsely covers an instance by adding one literal, but the same does not hold in the case of an uncovered instance. We account for this by adjusting SLS to remove all literals in a term that differ from an instance (line 22).

Any SLS algorithm starts by evaluating a random solution candidate. It then selects the next candidate from a neighborhood of the former candidate. This procedure is repeated until a solution is found. If no solution is found and no improvement is found for 600 steps, we restart the search with a different random formula. (These restarts did not seem to have an impact in the experiments we performed but may be significant in settings with larger step sizes or other datasets.) Therefore, one has to define a candidate space, a scoring function to evaluate a candidate solution, a neighborhood of a candidate solution, as well as a decision rule for selecting the next candidate out of a neighborhood. In SLS, the candidate space consists of all applicable k-term DNFs. The scoring function is defined as the number of misclassified instances by a given k-term DNF. The neighborhood of a candidate is given by all k-term DNFs that differ in one literal to the candidate. The next candidate is selected in accordance with a randomly drawn misclassified training instance (line 12). If the instance has a positive training label, with probability $p_{g1}$ a random term is modified (line 14), otherwise the term which differs least from the misclassified instance. With the probability $p_{g2}$ the modification is done by deleting a random literal (line 20). In the case of a negative training label (line 25), any term that covers the considered instance is chosen. In contrast to before, a literal not in accordance with the misclassified instance is added with a probability of $p_{s}$. Otherwise, a literal whose addition decreases the score over the training set most is appended. In the end, SLS returns the candidate that achieves the lowest score on the validation set.

Algorithm 3

1: **procedure** SLSearch$\left(k,maxIteration,p_{g1},p_{g2},p_{s},batchSize,training\;Stop,validation\;Set\right)$
2:**** formula←a randomly generated *k*-term DNF formula
3:**** optimalFormula←formula
4:**** iteration←0
5:**** minScore←∞
6: ** while** iteration<maxIteration and min Score>0 **do**
7:**** iteration←iteration+1
8:**** newScore←score(validationSet)
9: **  if** newScore<minScore **then**
10:**** minScore←newScore
11:**** optimalFormula←formula
12:**** missed Instance←random misclassified instance
13: **  if** missedInstancehas positive label **then**
14: **   with probability** $p_{g1}$
15:**** term←a term uniformly drawn from formula
16: **   otherwise**
17**:** term←the term in formula that differs in the smallest
18: **     **number of literals from missedInstance
19: **   with probability** $p_{g2}$
20:**** literals←a literal uniformly drawn from term
21: **   otherwise**
22:**** literals←all literals in term that differ from
23:**** missedInstance
24:**** formula←formula with literals removed from term
25: **  else if** missedInstancehas negative label **then**
26:**** term←a term in formulathat covers missed Instance
27: **   with probability** $p_{s}$
28:**** literal←a literal uniformly drawn from all possibilities
29: **   otherwise**
30:**** batch←uniformly pick batch Size many training instances
31:**** literal←a literal whose addition to term reduces
32:**** score(batch)the most
33:**** formula←formulawith literal added to term
** return** optimalFormula

## 4 Experimental Evaluation

The code for the following tests can be found on github.^5^ The parameters $p_{g1}$, $p_{g2}$ and $p_{s}$ are set to 0.5 for maximal randomness. For the SLS algorithm, we set k=40. In our experiments, we did not see any improvement for higher values of *k*. More details about the experiment parameters are given in Tables 6–8 in the Supplementary Appendix. We perform one-against-all testing so that the ground-truth-labels, which encode the classes of the data as one-hot vectors, are mapped to two classes. One class contains all images with the searched label, the other class contains all other labels. To prevent the neural network from predicting only the majority label, we balanced the labels in the training and test datasets such that one class comprises half of the dataset and the rest of the classes are randomly sampled so that each class is equally represented. We use three commonly used datasets with their predefined train-test splits: MNIST, FASHION-MNIST, and CIFAR10. For each dataset we used 5,000 samples as a holdout set for early stopping of the network and the rest for training. Each dataset has a designated test set with 10,000 samples. The dataset statistics are summarized in Table 1. More detailed information on the datasets can be found in Supplementary Appendix A.

**TABLE 1:** Statistics of the datasets.

Dataset	MNIST	FASHION-MNIST	CIFAR
Number of elements (training set)	60,000	60,000	50,000
Number of elements (test set)	10,000	10,000	10,000
Number of categories	10	10	10
Size of images	28 × 28 × 1	28 × 28 × 1	32 × 32 × 3

### 4.1 Deep Convolutional DNF Learner–Similarity

**FIGURE 2:**
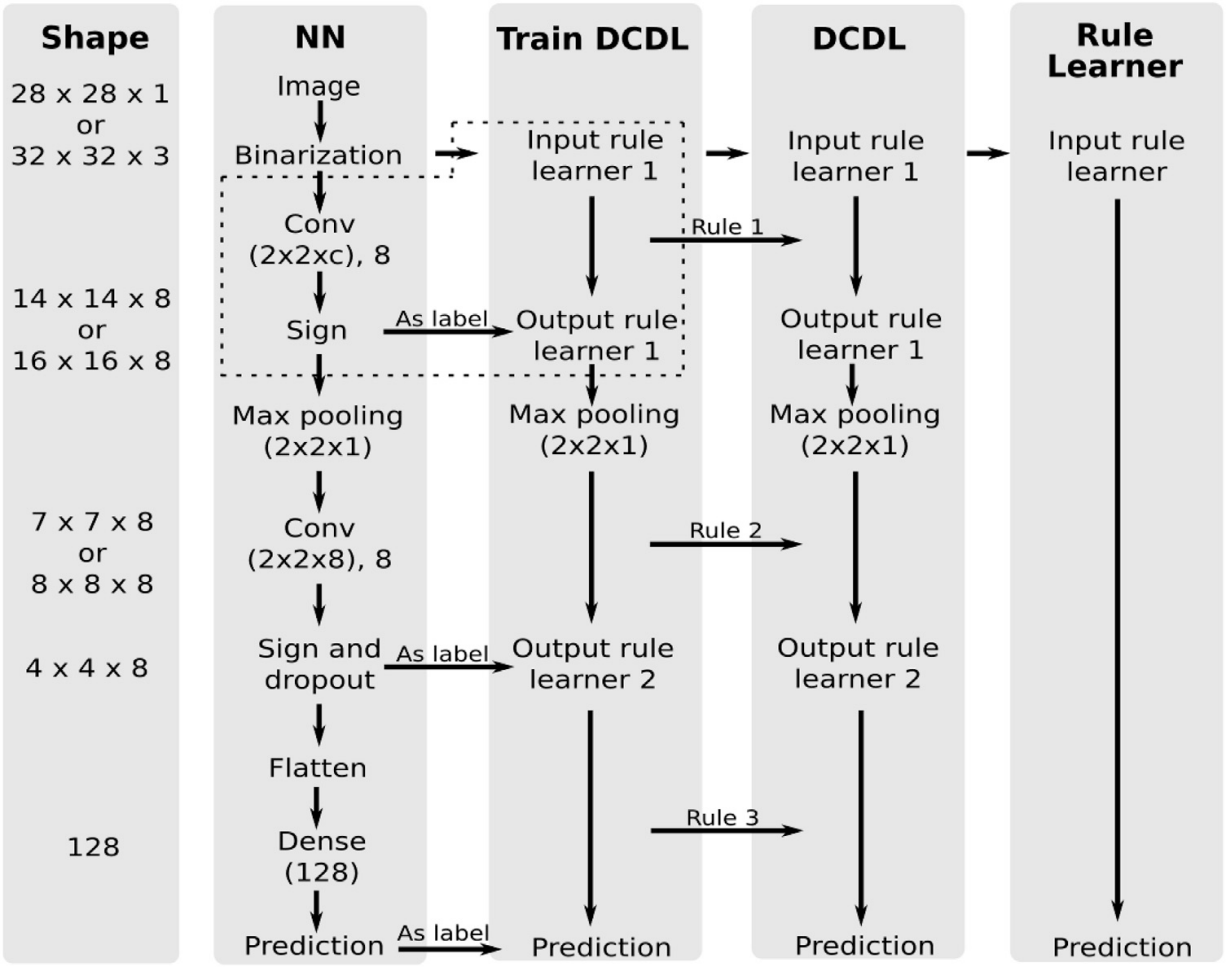
Experimental setup for comparing the neural network, the DCDL with a rule learner and the vanilla rule learner approach. *c* depends on the dataset and is the number of color channels. The content of the dotted box is shown in more detail in Figure 3.

In this section we compare our DCDL approach with SLS against the vanilla SLS algorithm and the DCDL approach with the decision tree rules learner against the vanilla decision tree rule learner. We look at their ability to model the behavior of a multilayer neural network for the datasets MNIST, FASHION-MNIST and CIFAR10. An overview of the experimental setup is given in Figure 2.

First a neural network is trained, which consists of two convolutional layers followed by a max pooling layer and a sign layer. The last layer is a dense layer with dropout. As soon as the neural net is trained, the output of the sign layers is used as a label for the training of DCDL. The sign layers transform the outputs to binary values for the rule learner. The two convolutional layers and the dense layer are each approximated with Boolean formulas, which are generated by the rule learner. The dithered images are initially used as input to the rule learner. After the first rule learner, the input in the following rule learner runs is the output of the previous formula. The intermediate results of the NN serve as labels.

**FIGURE 3:**
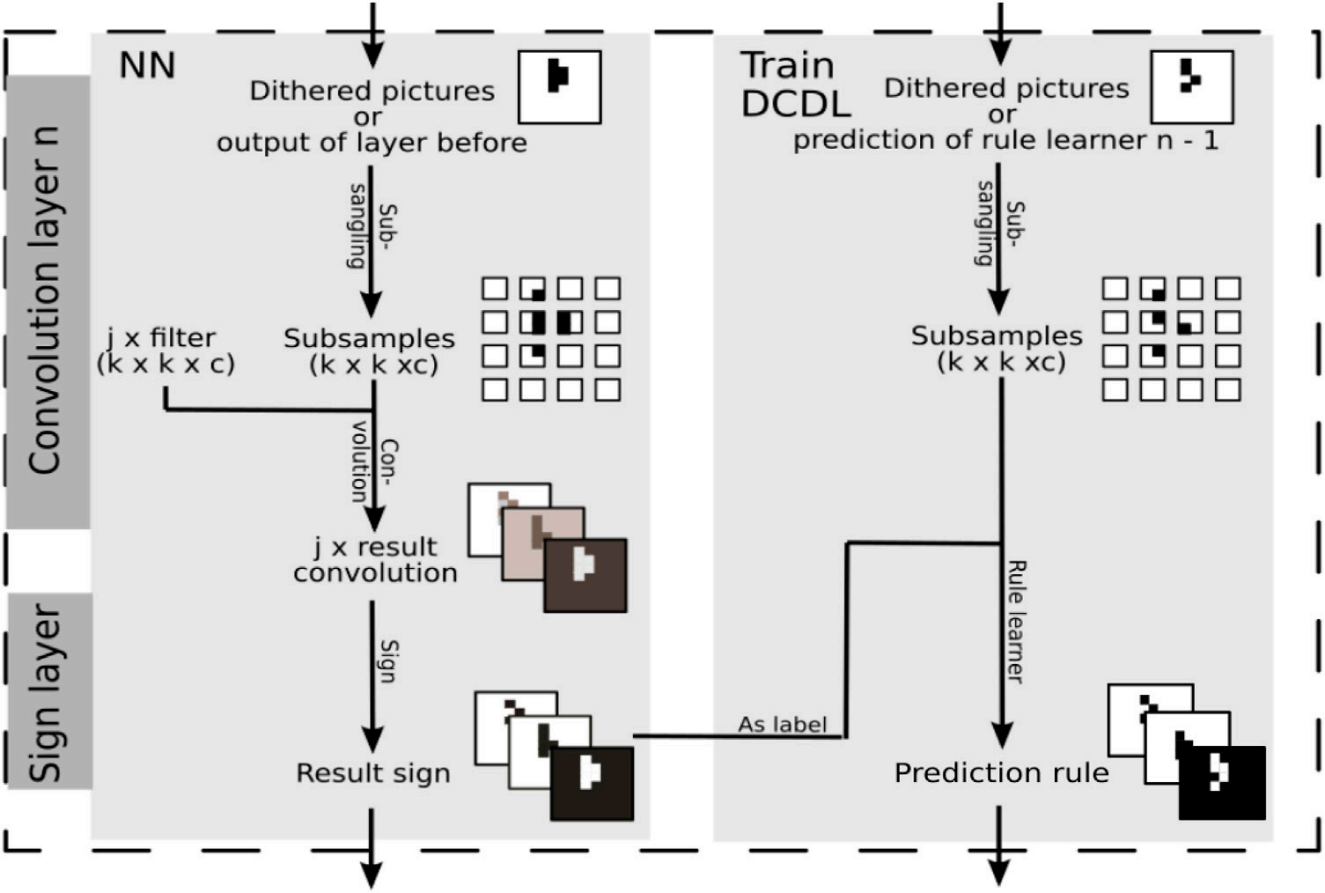
The approximation of a convolutional layer by the SLS algorithm. It is part of the whole experimental setup in Figure 2 as shown by the dotted box.

The approximation of the convolutional operation is, in contrast to the dense layer, not straightforward, so we will explain this process in detail here. Figure 3 shows the process graphically. In a convolutional layer, the input images are subsampled, and the samples are processed with the learned filters. Each sample is mapped to a value. This mapping creates a new representation of the images. Each filter gives its own representation. They are stacked as different channels. With the help of the sign layer, the representations are mapped to binary values.

The process of subsampling also takes place for the input of the DCDL approach. These samples are the input for the rule learner. As labels serves the channel output of the sign layer belonging to the filter which is being approximated with the help of the rule learner. Thus, each filter will be approximated by a logical formula. Using this procedure, DCDL approximates the operation of the NN with Boolean formulas.

In the vanilla SLS approach, only the input images and the corresponding label predicted by the NN are provided to the algorithm. The architecture of the NN and its functionality are not taken into account. It is evaluated using two different methods. In the prediction approach, the prediction of the neural network is used as a label for training. In the true label approach, the true labels of the images are used for training.

**FIGURE 4:**
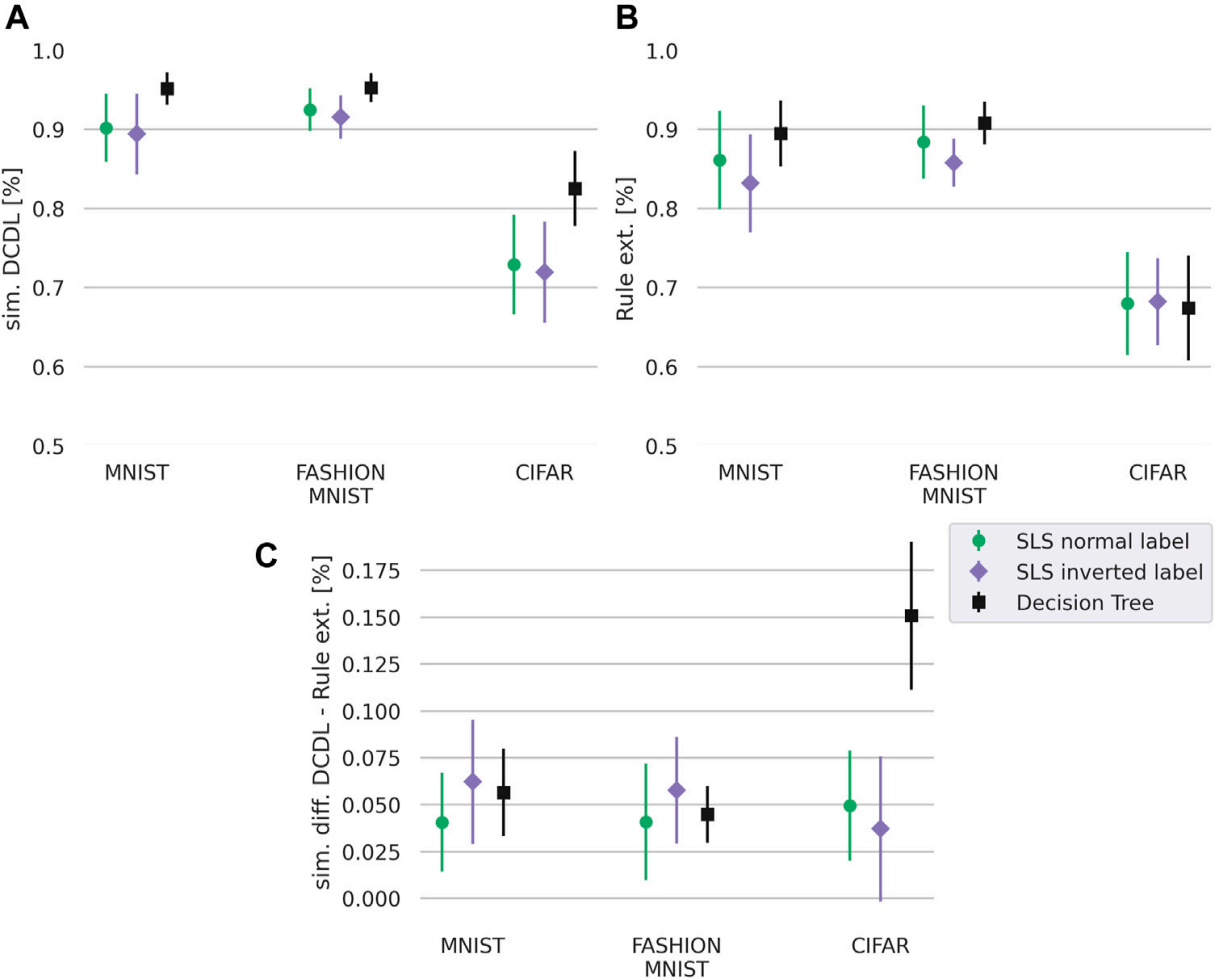
Similarity of the DCDL **(A)** and rule learning approaches **(B)** where for SLS we set k=40. Difference in similarity between both approaches **(C)**. The similarity is calculated with respect to the prediction of the neural network on the test set. The DCDL approach has higher similarity across all three datasets. The standard deviation is calculated from 30 runs overall, 3 for each class. The numeric values are given in Table 9 in the Supplementary Appendix.

We first focus on the question whether DCDL can better approximate the prediction of the neural network than the non-decompositional rule learning approaches. Our results in Figure 4 show that DCDL outperforms the non-decompositional rule learning approach on all three datasets and has the biggest advantage on the most complex dataset, CIFAR.

To calculate the similarity of the labels predicted by the neural network with the labels predicted by the rule learner, we calculate

$$sim=\frac{\sum_{i=1}^{n}1\left[y{\text{′}}_{i}=y{\text{″}}_{i}\right]}{n}$$ (5)

with *n* as the number of labels, 1 the indicator function, $y{\text{′}}_{i}$ as the prediction of the investigated approach, and $y{\text{″}}_{i}$ as the label calculated by the neural network.

### 4.2 Deep Convolutional DNF Learner—Accuracy

The above section shows that our method performs well in terms of similarity with the neural network. However, clearly, similarity does not necessarily correlate with accuracy. For example, it would be possible that the rule learner only models the errors that the network makes, leading to a high similarity but a bad performance on the actual labels. Therefore, we also compare the accuracy on the true labels of the predictions for the methods DCDL, vanilla rule learner (SLS or decision tree), and the neural network. For the vanilla rule learning algorithm, we differentiate between the method that was trained on the labels as predicted by the NN (rule learner prediction) and the method that was trained on the true labels (rule learner label). Again we use Eq. 5 to calculate the accuracy, but use the true labels of the test data instead of the labels predicted by the neural network.

**FIGURE 5:**
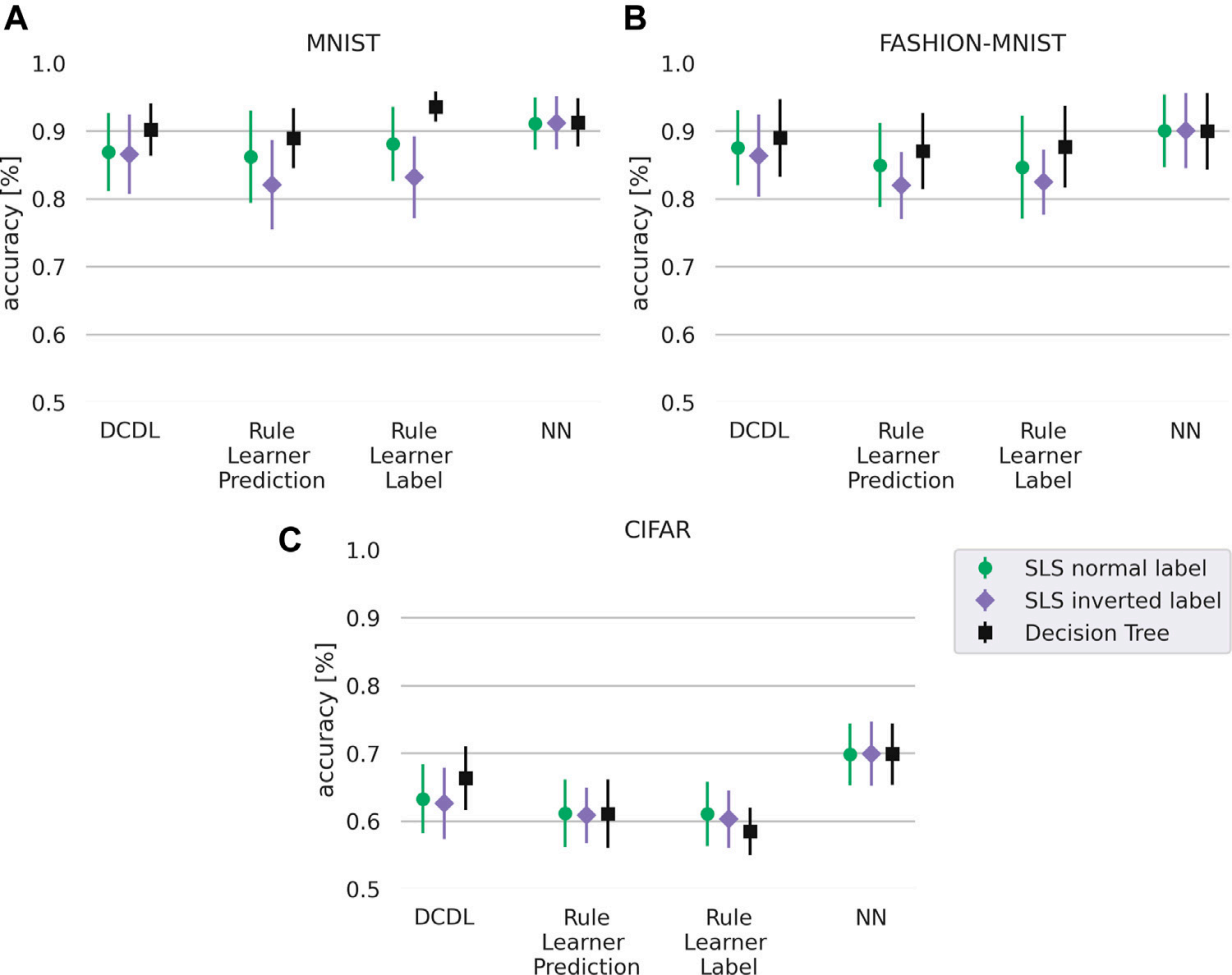
Accuracy for different datasets with normal label and inverted label of our DCDL approach, a rule learner, a rule learner with true labels, and the neural network. As rule learners we compare SLS with k=40 and decision trees. **(A)**: MNIST, **(B)** FASHION-MNIST, **(C)**: CIFAR. The standard deviation is calculated from 30 runs overall, 3 for each class. The numeric values are given in Table 10 in the Supplementary Appendix.

Our results in Figure 5 show the performance on the task of learning to predict the label and show that the neural network outperforms the other methods. DCDL and the non-decompositional rule learning approaches SLS and Decision Tree perform at a comparable level except for the CIFAR dataset, where DCDL has a slight advantage over the non-decompositional rule learning methods as also shown by the significance values in Table 2. Here, we also evaluated the statistical significance using a corrected resampled *t*-test (Nadeau and Bengio, 2003) with α=0.05. The null-hypothesis is that the different groups perform equally. For all datasets, the rule learning approaches perform worse than the neural network. This shows that the approximation by our rule learner is not perfect and we hypothesize that improving the SLS algorithm or replacing it with a different rule learner could remedy this.

**TABLE 2:** Shown are *p*-values of the corrected resampled *t*-test (Nadeau and Bengio, 2003) for MNIST, FASHION-MNIST and CIFAR with SLS normal labels for the accuracy values plotted in Figure 5. Gray-shaded are the pairs for which the null hypothesis is rejected with significance level α=0.05 using a corrected resampled *t*-test (Nadeau and Bengio, 2003).

	Rule learner prediction	Rule learner label	Neural network
MNIST			
DCDL	0.52	0.38	0.00
Rule learner prediction	—	0.09	0.00
Rule learner label	—	—	0.02
FASHION-MNIST			
DCDL	0.03	0.10	0.00
Rule learner prediction	—	0.81	0.00
Rule learner label	—	—	0.01
CIFAR			
DCDL	0.08	0.04	0.00
Rule learner prediction	—	0.90	0.00
Rule learner label	—	—	0.00

To test this hypothesis we performed the same experiment with a different rule learner, a decision tree instead of the SLS. The result is shown in Figure 5 with corresponding significance values in Table 3. The decision tree is able to achieve a higher accuracy than the SLS algorithm. However, we note that the decision tree rule learner is a heuristic rule learning approach that relies on pruning whereas the SLS learner is theoretically well founded and adjustable in the parameter *k*. Higher values of the parameter *k* allow us to achieve better visualizations. While the rules with the SLS rule learner are less discriminative and achieve a lower accuracy, they are able to learn descriptive rules as shown in the next section. Overall, Figure 5 shows that DCDL is still superior to the vanilla rule learners in approximating the NN, even if the rule learner is changed.

**TABLE 3:** Shown are *p*-values of the corrected resampled *t*-test (Nadeau and Bengio, 2003) for MNIST, FASHION-MNIST and CIFAR with decision tree normal labels for the accuracy values plotted in Figure 5. Gray-shaded are the pairs for which the null hypothesis is rejected with significance level α=0.05 using a corrected resampled *t*-test (Nadeau and Bengio, 2003).

	Rule learner prediction	Rule learner label	Neural network
MNIST			
DCDL	0.00	0.00	0.01
Rule learner prediction	—	0.00	0.00
Rule learner label	—	—	0.04
FASHION-MNIST			
DCDL	0.00	0.06	0.00
Rule learner prediction	—	0.24	0.00
Rule learner label	—	—	0.00
CIFAR			
DCDL	0.00	0.00	0.00
Rule learner prediction	—	0.06	0.00
Rule learner label	—	—	0.00

In addition to heuristic rule learners such as decision trees and approximate but well founded methods like SLS, another line of research develops exact and theoretically well founded rule learners (Demirović and Stuckey, 2020; Yu et al., 2020; Ignatiev et al., 2021). These models do not compare favorably to SLS in terms of runtime which does not make them a suitable choice in our model.

In Figure 5 we also evaluate the task of learning the inverse of the label which makes a difference in the case of the logic-based approaches. It can be seen as a harder task as the approaches need to learn what e.g. a one is *not*, instead of learning what a one is. Here, DCDL clearly outperforms the non-decompositional rule learning approach on the MNIST and FASHION-MNIST datasets and still provides a slight advantage on the CIFAR dataset, which also can be verified in Table 4. The poor performance of the classifiers on the CIFAR dataset is most likely partly caused by the dithering. The network architecture might also play a role. Overall we conclude from the results that on more complex tasks, the DCDL has an advantage over the non-decompositional rule learning methods.

**TABLE 4:** Shown are *p*-values of the corrected resampled *t*-test (Nadeau and Bengio, 2003) for MNIST, FASHION-MNIST and CIFAR with SLS inverted labels for the accuracy values plotted in Figure 5 Gray-shaded are the pairs for which the null hypothesis is rejected with significance level α=0.05 using a corrected resampled *t*-test (Nadeau and Bengio, 2003).

	Rule learner prediction	Rule learner label	Neural network
MNIST			
DCDL	0.00	0.01	0.00
Rule learner prediction	—	0.18	0.00
Rule learner label	—	—	0.00
FASHION-MNIST			
DCDL	0.00	0.01	0.00
Rule learner prediction	—	0.59	0.00
Rule learner label	—	—	0.00
CIFAR			
DCDL	0.16	0.09	0.00
Rule learner prediction	—	0.55	0.00
Rule learner label	—	—	0.00

### 4.3 Visualization of Logical Formulas

We already showed an example for the visualization of a simple formula in Section 3.1.1. Now, we want to look at the visualization of more complex formulas that are found by our algorithm. If the rule search is conducted with a small *k*, the visualized rules tend to be discriminative and often highlight only a single pixel, thus making them hard to interpret. To counter this, we set *k* to higher values in order to learn rules that are more characteristic. However, when visualizing *k* predicates for high *k*, this produces too many images to consider them individually. Therefore, we add a reduce step that sums the visualization of all formulas pixel-wize to one image *X*. Afterward *X* is scaled to the range −1 to 1 with .

$$scaling\left(x,X\right)=\{\begin{array}{cc} -\frac{x}{min\left(X\right)}, & \text{for }x<0\\ \frac{x}{max\left(X\right)}, & \text{for }x\geq 0 \end{array}$$ (6)

**FIGURE 6:**
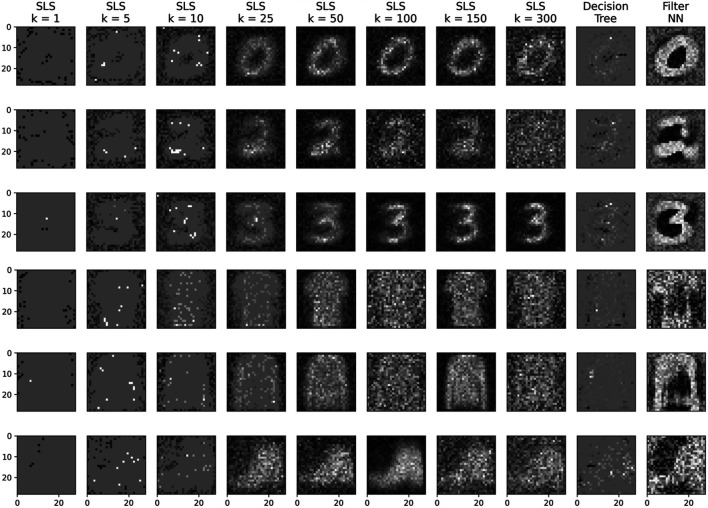
A visualization of the rules learned by the SLS algorithm (k=[1,5,10,25,50,100,150,300]), decision tree and the filter used in the NN. Rows 1–3 are the rules for labels [Zero, Two, Three] of the MNIST dataset. Row 4–6 are the rules for the labels [T-shirt/Top, Sweater, Ankleboot] of the FASHION-MNIST dataset. For small *k*, discriminative rules are learned, and for large *k* characteristic rules. In rows 2 and 5 for k=300 it can be seen how disjunctions, which were not further optimized after initialization, overlay the visualization of the optimized disjunctions.

As Figure 6 shows, this leads to visualizations that look almost like probability densities or prototypes. The comparison to the convolutional filters shows that this procedure leads to good visualizations that are comparable to the convolutional filters. The influence of different settings of the parameter *k* and results for several labels of FASHION-MNIST are shown in Figure 6.

Comparing the visualization of DCDL in Figure 6 with the visualization of the rules learned by the decision tree shows that the decision tree, as it presumable learns more discriminative rules, has visualizations that are, subjectively, harder to interpret. Therefore, while the decision tree learns rules with higher accuracy as discussed in the last section, it is less well suited to the visualization of the rules. The SLS however, allows to construct characteristic rules by increasing parameter *k* without affecting the accuracy (as shown in Figure 8 in the Supplementary Appendix).

**FIGURE 7:**
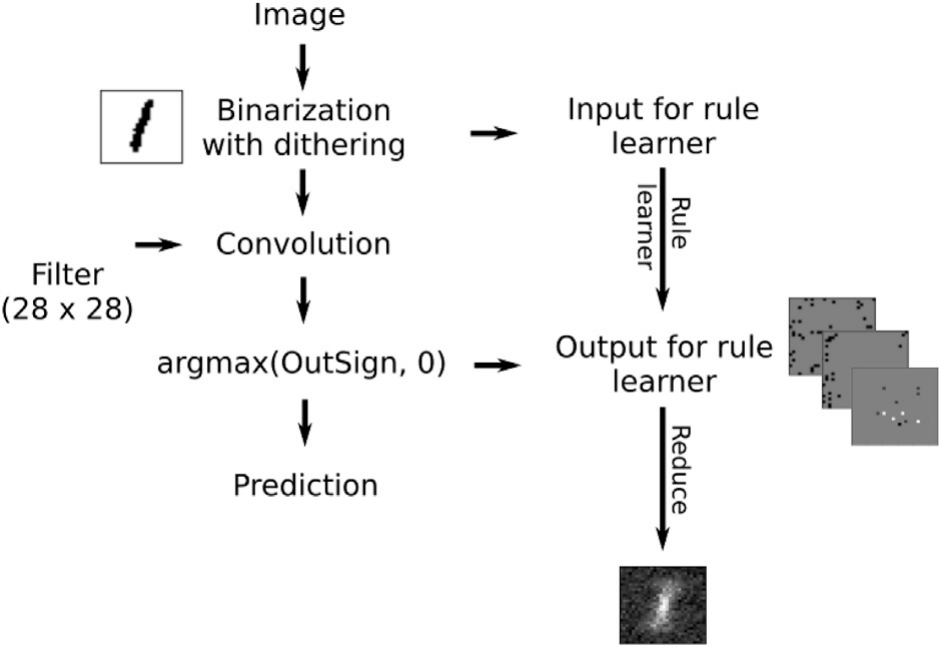
The test setup for approximating convolution operations with logical formulae.

The architecture of the neural network is shown in Figure 7. It consists of a convolutional layer followed by a sign layer and a dense layer. The dense layer converts the scalar output of the sign layer into a one-hot vector. The weights of the dense layer were set to [1,0]. The dithered images are the input for the SLS algorithm and the output of the sign layer is the label for the SLS algorithm. The visualization in Figure 6 was done on the MNIST dataset with a filter size that is equal to the size of the image. Note that for the case of MNIST, smaller filter sizes do not result in interpretable visualizations. However, in principle we can also choose filter sizes much smaller than the image itself if the images consist of complex scenes where the number itself is only a small part of the image for example. The selection of a filter size that leads to an interpretable visualization is left for future work.

We would like to emphasize that our goal is not to show that our rules lead to more interpretable visualizations than that of the filters itself. The comparison shows however, that the visualization of our rules leads to an image which is closer to a density rather than a flat pattern. The fact alone that we are able to visualize logical rules for images in this way, is a novel contribution in our view. This is in addition to other advantages that logical rules provide, which are not present in convolutional filters. To sum up, we showed with the help of a simple example how the individual predicates as well as the complete convolutional rule may be visualized.

## 5 Conclusion

We investigated how convolutional rules enable the extraction of interpretable rules for images from binary neural networks. We showed the successful visualization by means of an example. Additionally, the similarity to the functionality of the neural network was measured on three different datasets and found to be higher for the decompositional approach than the non-decompositional rule learning approach. We think there is potential in decompositional approaches for the extraction and visualization of characteristic rules. Although the logical formulas are large for human visual inspection on real-world data, their representation makes deep learning models, in principle, amenable to formal verification and validation.

In future research, we aim to incorporate further state-of-the-art components of NNs while preserving the ability of the network to be transformed into (convolutional) logical rules. Our work suggests that the combination of binary NNs and k-DNF is promising combination. To this end, one should develop a differentiable version of DCDL based on, e.g., differentiable sub modular maximization (Tschiatschek et al., 2018) or differentiable circuit SAT (Powers et al., 2018). Generally, one should explore DCDL as a new perspective on neuro-symbolic AI (Garcez and Lamb, 2020).

## Data Availability

Publicly available datasets were analyzed in this study. This data can be found here: http://yann.lecun.com/exdb/mnist/, https://github.com/zalandoresearch/fashion-mnist, https://www.cs.toronto.edu/∼kriz/cifar.html.

## Footnotes

1 This paper is an extension of three workshop papers that were presented at the DeCoDeML workshops at ECML/PKDD 2019 and 2020: Sophie Burkhardt, Nicolas Wagner, Johannes Fürnkranz, Stefan Kramer: Extracting Rules with Adaptable Complexity from Neural Networks using K-Term DNF Optimization; Nicolas Wagner, Sophie Burkhardt, Stefan Kramer: A Deep Convolutional DNF Learner; Sophie Burkhardt, Jannis Brugger, Zahra Ahmadi and Stefan Kramer: A Deep Convolutional DNF Learner.

2 Implemented in https://python-pillow.org/.

3 Based on https://github.com/hbldh/hitherdither, we also used the given error diffusion matrix.

4
https://github.com/kramerlab/DCDL

5
https://github.com/kramerlab/DCDL
