# Supplementary material for: Rule Extraction From Binary Neural Networks With Convolutional Rules for Model Validation
Source: Front Artif Intell. 2021 Jul 21;4:642263. doi: 10.3389/frai.2021.642263 (PMC8336635; doi:10.3389/frai.2021.642263)
Supplement: Supplementary file 1 [file DataSheet1.pdf]

Appendix A Summary tables for the datasets

Table 1: This table summarizes the statistics of the datasets.

Label	0	1	2	3	4	5	6	7	8	9
					MNIST					
Categories	Zero	One	Two	Three	Four	Five	Six	Seven	Eight	Nine
Number of elements (training set)	5923	6742	5958	6131	5842	5421	5918	6265	5851	5949
Number of elements (test set)	980	1135	1032	1010	982	892	958	1028	974	1009
					FASHION-MNIST					
Categories	T-shirt /Top	Trou- ser	Pull- over	Dress	Coat	Sandal	Shirt	Sneaker	Bag	Ankle boot
Number of elements (training set)	6000	6000	6000	6000	6000	6000	6000	6000	6000	6000
Number of elements (test set)	1000	1000	1000	1000	1000	1000	1000	1000	1000	1000
					CIFAR					
Categories	air- plane	Auto- mobile	Bird	Cat	Deer	Dog	Frog	Horse	Ship	Truck
Number of elements (training set)	5000	5000	5000	5000	5000	5000	5000	5000	5000	5000
Number of elements (test set)	1000	1000	1000	1000	1000	1000	1000	1000	1000	1000

A.1 MNIST

The MNIST dataset (Lecun et al. 1998) consists of 60,000 training images and 10,000 test images of written numbers. Each image is 28x28 pixel and has 1 color channels. There are 10 categories. The distribution of images among the 10 categories is given in Table 5.

A.2 FASHION-MNIST

The FASHION-MNIST dataset (Xiao et al. 2017) consists of 60,000 training images and 10,000 test images of fashion categories. Each image is 28x28 pixel and has 1 color channel. There are 10 categories. The distribution of images among the 10 categories is given in Table 5.

A.3 CIFAR

The CIFAR dataset (Krizhevsky 2012) consists of 50,000 training images and 10,000 test images. Each image is 32x32 pixel and has 3 color channels. There are 10 categories. The distribution of images among the 10 categories is given in Table 5.

Appendix B Parameters of the experiments

All parameters needed to perform the experiments are listed in the settings files in the github repository of this paper. The most important parameters are briefly presented below.

B.1 DCDL-Accuracy and DCDL-Similarity

The parameters for the DCDL-Accuracy (4.2) and DCDL-Similarity (4.1) experiment are the same. The parameter for the NN are listed in Table 2. As activation function in the forward pass we use $a(x) = sign(sign(x) - 0.1)$ so that the activation evaluates to -1 when $x \leqq 0$ and to 1 when $x > 0$. In the backward pass we overwrite the gradient of the activation with the identity.
The parameters of the SLS algorithm are listed in Table 3. For the vanilla SLS algorithm and to approximate the layers of the neural network, the same parameters are used.

Table 2: This table lists the parameters of the neural network experiments for measuring the accuracy.

Category	Value
Training steps	2000
Learning rate	10^{-3}
Dropout rate	0.4
Shape filter convolution	(2x2x number output channel of layer before)
Number filter	8
Stride convolution	2
Batch size	2^{10}
Check interval at validation set	25
Shape max pooling layer	(2,2)
Max pooling stride	2

Table 3: This table lists the parameters of the SLS algorithm for the accuracy measurements.

Category	Value
Steps	2000
k	40
Batch size	2^{10}
p_{g1}	0.5
p_{g2}	0.5
p_{g3}	0.5
Batch size	2^{10}
Steps without change before restart	600
Initialization start formula	random

B.2 Visualization of Logical Formulas

The parameters for visualization experiment in Section 4.3 which differ compared to Table 2 and Table 3 are given in Table 4.

Table 4: This table lists the parameters for the experiments on the visualization of logical formulas.

Category	Value
Steps NN	200
Filter size NN	(28,28)
Batch size NN	2^{14}
k	[1,5,10,25,50,100,150,300]

Appendix C Exact Values from Plots

Appendix D Influence of Parameter k

Table 5: This table shows the exact values plotted in Figure 4 (similarity) of the paper.

	DCDL	Rule learner	DCDL - Rule learner
		SLS normal label	
MNIST	0.90 ± 0.04	0.86 ± 0.06	0.04 ± 0.03
FASHION-MNIST	0.93 ± 0.03	0.88 ± 0.05	0.04 ± 0.03
CIFAR	0.73 ± 0.06	0.68 ± 0.07	0.05 ± 0.03
		SLS inverted label	
MNIST	0.89 ± 0.05	0.83 ± 0.06	0.06 ± 0.03
FASHION-MNIST	0.92 ± 0.03	0.86 ± 0.03	0.06 ± 0.03
CIFAR	0.72 ± 0.06	0.68 ± 0.05	0.04 ± 0.04
		Decision tree	
MNIST	0.95 ± 0.02	0.90 ± 0.04	0.06 ± 0.02
FASHION-MNIST	0.95 ± 0.02	0.91 ± 0.03	0.04 ± 0.02
CIFAR	0.83 ± 0.05	0.67 ± 0.07	0.15 ± 0.04

Table 6: This table shows the exact values of SLS normal label, SLS inverted label, and the decision tree plotted in Figure 5 of the paper.

	DCDL	Rule learner prediction	Rule learner label	NN
		SLS normal label		
MNIST	0.87 ± 0.06	0.86 ± 0.07	0.88 ± 0.05	0.91 ± 0.04
FASHION-MNIST	0.88 ± 0.06	0.85 ± 0.06	0.85 ± 0.08	0.90 ± 0.05
CIFAR	0.63 ± 0.05	0.61 ± 0.05	0.61 ± 0.05	0.70 ± 0.05
		SLS inverted label		
MNIST	0.87 ± 0.06	0.82 ± 0.07	0.83 ± 0.06	0.91 ± 0.04
FASHION-MNIST	0.86 ± 0.06	0.82 ± 0.05	0.82 ± 0.05	0.90 ± 0.06
CIFAR	0.63 ± 0.05	0.61 ± 0.04	0.60 ± 0.04	0.70 ± 0.05
		Decision tree		
MNIST	0.90 ± 0.04	0.89 ± 0.04	0.94 ± 0.02	0.91 ± 0.04
FASHION-MNIST	0.89 ± 0.06	0.87 ± 0.06	0.88 ± 0.06	0.90 ± 0.06
CIFAR	0.66 ± 0.05	0.61 ± 0.05	0.58 ± 0.03	0.70 ± 0.05

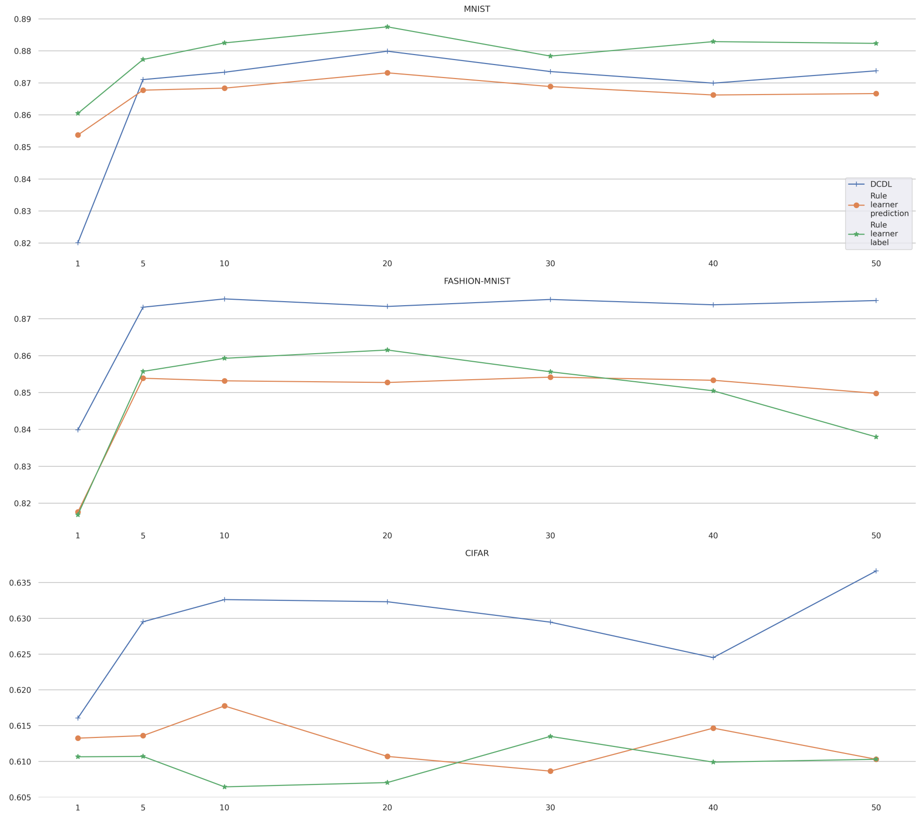

Figure 1: This figure shows the influence of the parameter k on the accuracy of the DCDL and the vanilla SLS approaches. k was chosen from the values $[1, 5, 10, 20, 30, 40, 50]$. The top plot is for MNIST, the middle plot is for FASHION-MNIST and the bottom plot is for CIFAR. For each value of k, the mean was calculated over all labels in a dataset. For $k \geq 5$ and above, no improvement can be observed for larger k.
